# The genome sequence of the Plain Longtail butterfly, *Spicauda simplicius* (Stoll, 1807)

**DOI:** 10.12688/wellcomeopenres.22457.1

**Published:** 2024-06-13

**Authors:** Pedro Ribeiro, Pável Matos-Maraví, Daniel Linke, Joana Meier, Wellcome Sanger Institute Tree of Life Management, Samples and Laboratory team, Wellcome Sanger Institute Scientific Operations: Sequencing Operations, Wellcome Sanger Institute Tree of Life Core Informatics team, Tree of Life Core Informatics collective

**Affiliations:** 1 Biology Centre of the Czech Academy of Sciences, Institute of Entomology, České Budějovice, Czech Republic; 2 Faculty of Science, University of South Bohemia, České Budějovice, Czech Republic; 3 Wellcome Sanger Institute, Hinxton, England, UK

**Keywords:** Spicauda simplicius, Plain Longtail butterfly, genome sequence, chromosomal, Lepidoptera

## Abstract

We present a genome assembly from an individual female *Spicauda simplicius* (the Plain Longtail butterfly; Arthropoda; Insecta; Lepidoptera; Hesperiidae). The genome sequence is 610.1 megabases in span. Most of the assembly is scaffolded into 32 chromosomal pseudomolecules, including the Z and W sex chromosomes. The mitochondrial genome has also been assembled and is 15.54 kilobases in length. Gene annotation of this assembly on Ensembl identified 18,506 protein coding genes.

## Species taxonomy

Eukaryota; Opisthokonta; Metazoa; Eumetazoa; Bilateria; Protostomia; Ecdysozoa; Panarthropoda; Arthropoda; Mandibulata; Pancrustacea; Hexapoda; Insecta; Dicondylia; Pterygota; Neoptera; Endopterygota; Amphiesmenoptera; Lepidoptera; Glossata; Neolepidoptera; Heteroneura; Ditrysia; Obtectomera; Hesperioidea; Hesperiidae; Eudaminae; Eudamini; *Spicauda*; *Spicauda simplicius* (Stoll, 1807) (NCBI:txid355208).

## Background

**Figure 1.**
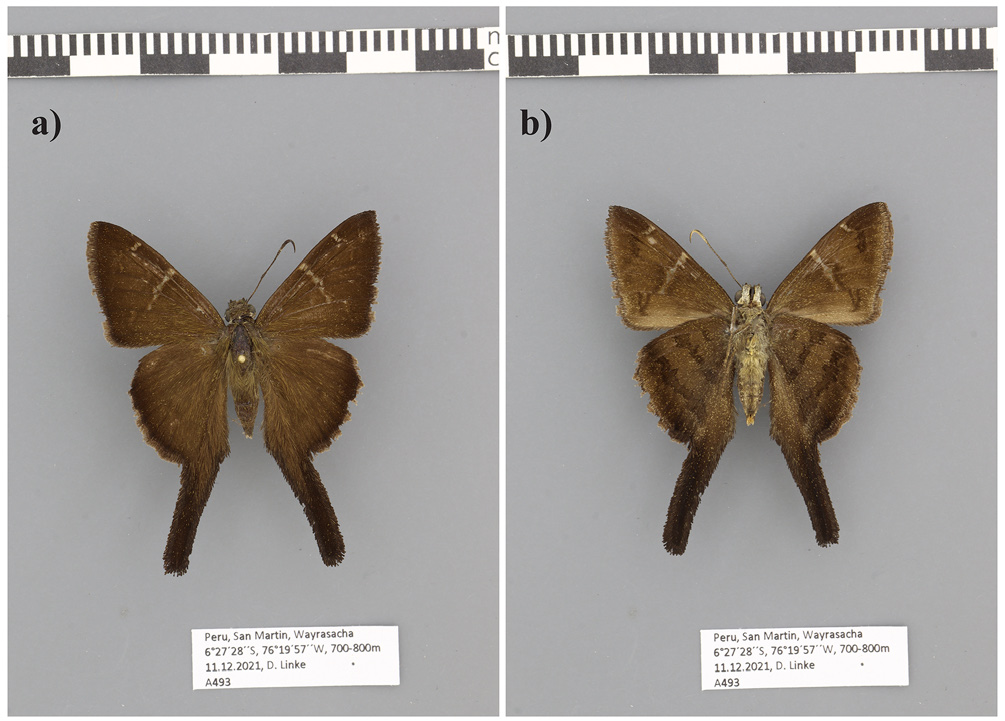
Photograph of a *Spicauda simplicius* specimen collected in the same locality as the sequenced specimen.

*Spicauda simplicius* (Plain Longtail) is a butterfly of the family Hesperiidae with a neotropical distribution, ranging from northern Mexico to northern Argentina ( Evans, 1951). The common name refers to its cryptic colouration (it is plain brown and lacks any white apical forewing patches found in congeneric species) and elongated hind wing tails ( Figure 1). The species commonly co-occurs in habitats with other widespread *Spicauda* species (e.g., *S. tanna*, *S. teleus*, *S. procne*). The species lacks any clearly defined sexual dimorphism.

*Spicauda simplicius* is a common species in disturbed environments, with strays being reported as far north as Texas ( Rickard, 1977) and a single individual being reported from California ( Tilden, 1976). It is absent from the Caribbean islands except Trinidad and Tobago ( Cock, 1982) and has recently become established in Grenada ( Lewis *et al.*, 2012), after being found only as an isolated individual ( Smith *et al.*, 1994).

The species utilises a wide array of habitats, but it prefers degraded or open habitats, although it can also be found in closed forests. As of 01/2024 the species has not been assessed by IUCN but will likely be of least concern having stable populations as it is highly abundant, is widespread and can be commonly found in degraded or urban environments during the whole year. Additionally, this species feeds on a variety of widespread Fabaceae (bean family). Hostplant records from Costa Rica (plant species = 20, *n* = 322) include *Arachis* L., *Calopogonium* Desv., *Centrosema* (DC.) Benth., *Desmodium* Desv., *Galactia* P. Browne, *Phaseolus* L., *Rhynchosia* Lour., *Teramnus* P. Browne and *Vigna* Savi ( Janzen & Hallwachs, 2024). Other hostplant records from the Neotropics include *Tipuana tipu* (Benth.) Kuntze (Brazil), *Glycine max* (L.) Merr. (Uruguay) and *Pueraria phaseoloides* (Roxb.) Benth. (Trinidad) (e.g. Beccaloni, 2019; Biezanko *et al.*, 1974; Cock *et al.*, 2015; da Lima & e Silva, 1967) .

Historically, the species has been placed in numerous different genera, i.e., *Papilio*, *Goniurus*, *Thymele*, *Eudamus*, *Urbanus*. The genus *Spicauda* Grishin was created in 2019 ( Li *et al.*, 2019). Its haploid chromosome number is 31 ( de Lesse, 1967).

## Genome sequence report

The genome was sequenced from one female *Spicauda simplicius* ( Figure 1) collected from Tarapoto, San Martin, Peru (–6.49, –76.36). A total of 23-fold coverage in Pacific Biosciences single-molecule HiFi long reads was generated. Primary assembly contigs were scaffolded with chromosome conformation Hi-C data. Manual assembly curation corrected 97 missing joins or mis-joins and removed 21 haplotypic duplications, reducing the assembly length by 1.41% and the scaffold number by 14.78%.

**Figure 2.**
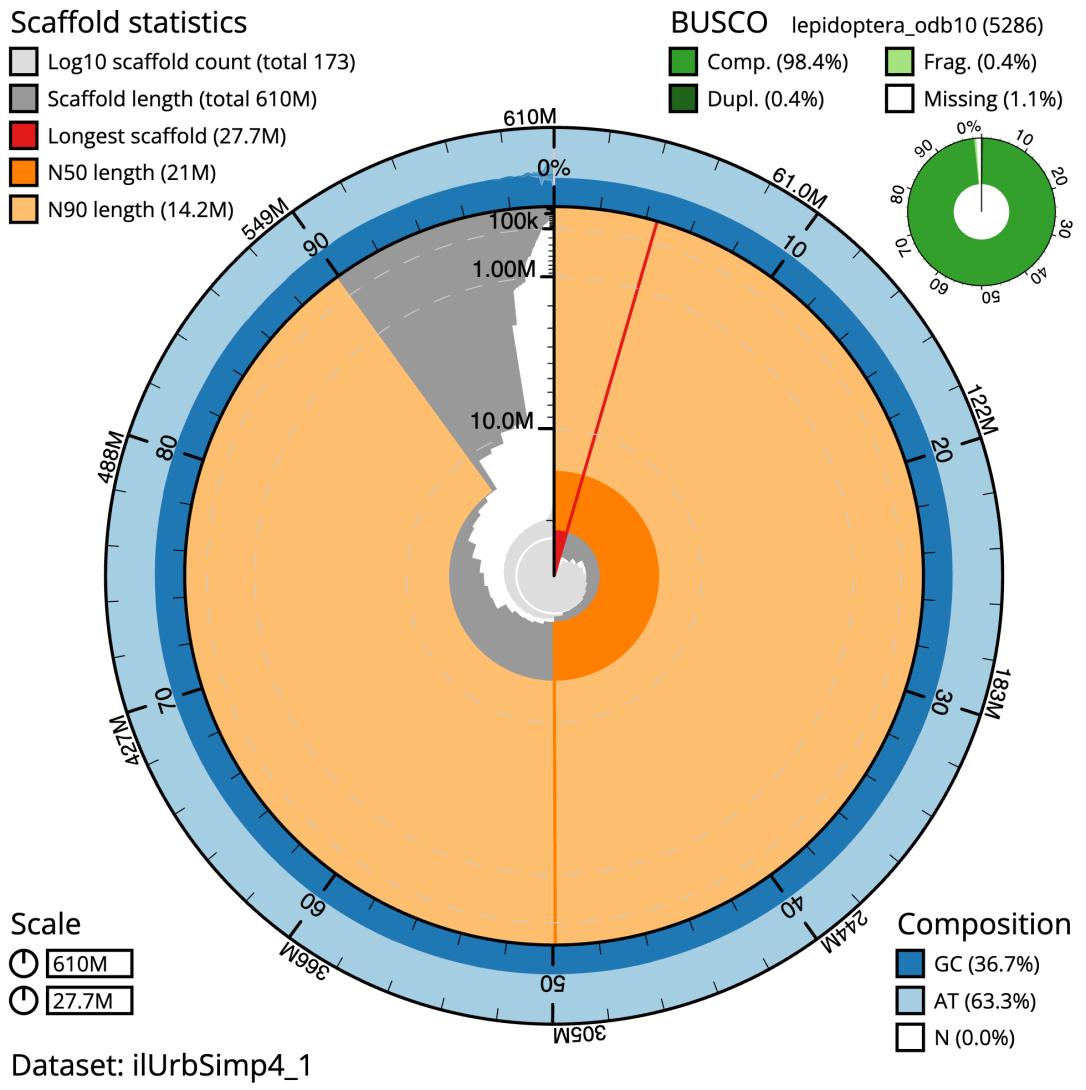
Genome assembly of *Spicauda simplicius*, ilUrbSimp4.1: metrics. The BlobToolKit snail plot shows N50 metrics and BUSCO gene completeness. The main plot is divided into 1,000 size-ordered bins around the circumference with each bin representing 0.1% of the 610,070,898 bp assembly. The distribution of scaffold lengths is shown in dark grey with the plot radius scaled to the longest scaffold present in the assembly (27,661,612 bp, shown in red). Orange and pale-orange arcs show the N50 and N90 scaffold lengths (21,027,237 and 14,173,452 bp), respectively. The pale grey spiral shows the cumulative scaffold count on a log scale with white scale lines showing successive orders of magnitude. The blue and pale-blue area around the outside of the plot shows the distribution of GC, AT and N percentages in the same bins as the inner plot. A summary of complete, fragmented, duplicated and missing BUSCO genes in the lepidoptera_odb10 set is shown in the top right. An interactive version of this figure is available at https://blobtoolkit.genomehubs.org/view/Urbanus%20simplicius/dataset/ilUrbSimp4_1/snail.

**Figure 3.**
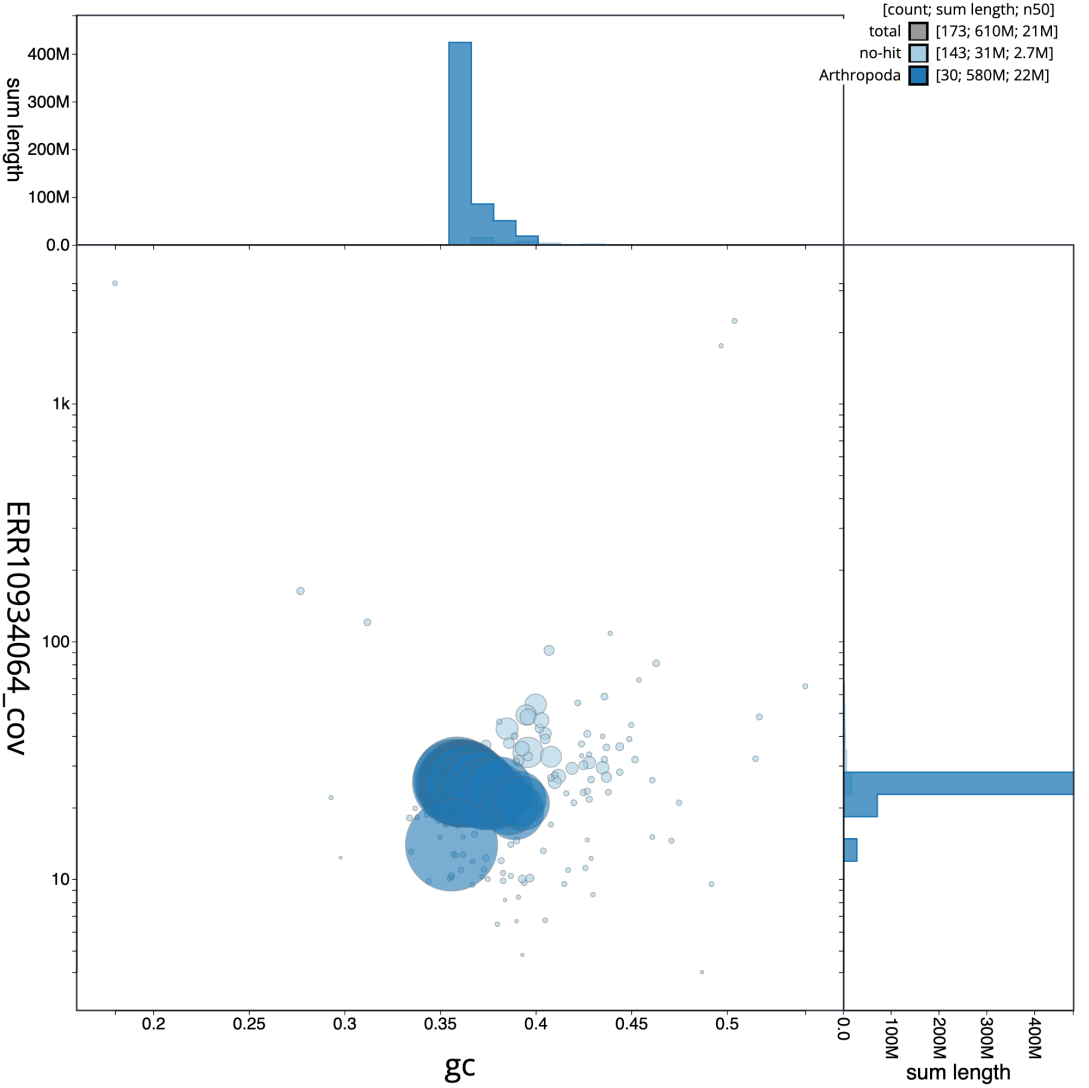
Genome assembly of *Spicauda simplicius*, ilUrbSimp4.1: BlobToolKit GC-coverage plot. Sequences are coloured by phylum. Circles are sized in proportion to sequence length. Histograms show the distribution of sequence length sum along each axis. An interactive version of this figure is available at https://blobtoolkit.genomehubs.org/view/Urbanus%20simplicius/dataset/ilUrbSimp4_1/blob.

**Figure 4.**
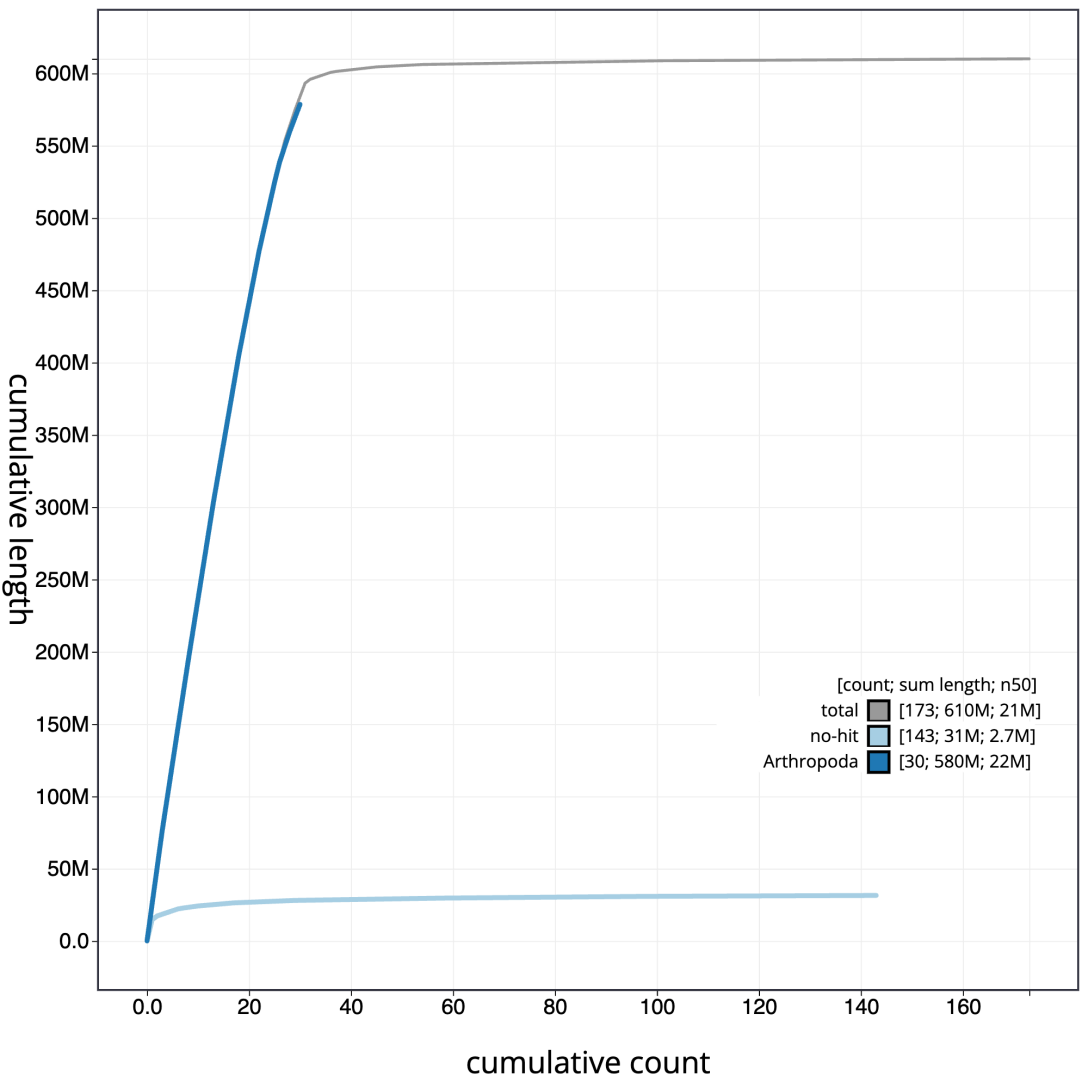
Genome assembly of *Spicauda simplicius*, ilUrbSimp4.1: BlobToolKit cumulative sequence plot. The grey line shows cumulative length for all sequences. Coloured lines show cumulative lengths of sequences assigned to each phylum using the buscogenes taxrule. An interactive version of this figure is available at https://blobtoolkit.genomehubs.org/view/Urbanus%20simplicius/dataset/ilUrbSimp4_1/cumulative.

**Figure 5.**
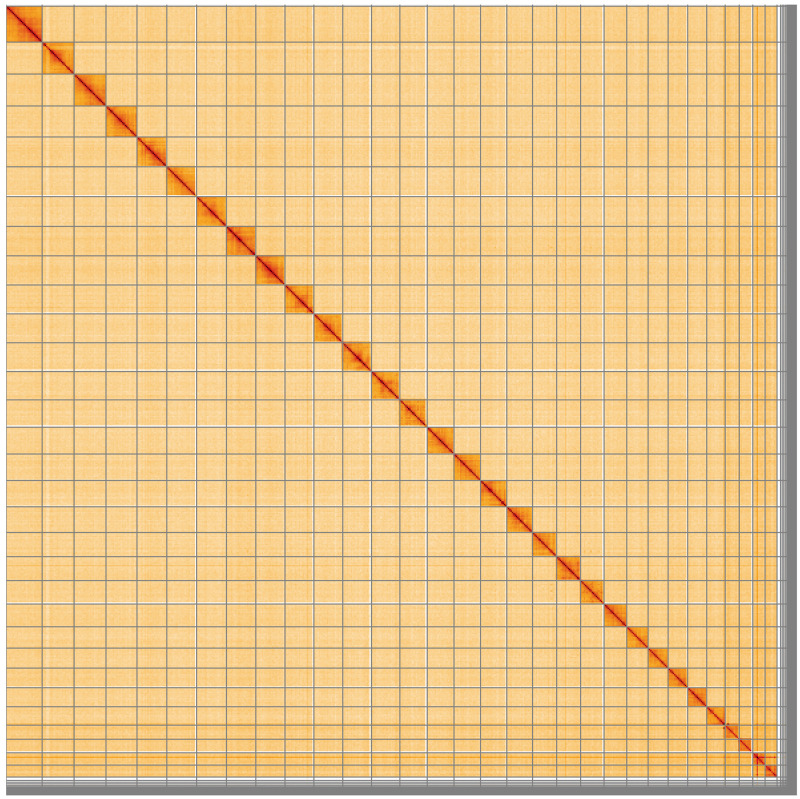
Genome assembly of *Spicauda simplicius*, ilUrbSimp4.1: Hi-C contact map of the ilUrbSimp4.1 assembly, visualised using HiGlass. Chromosomes are shown in order of size from left to right and top to bottom. An interactive version of this figure may be viewed at https://genome-note-higlass.tol.sanger.ac.uk/l/?d=dRd7w2eUTVmD4XwxPqvi8A.

The final assembly has a total length of 610.1 Mb in 172 sequence scaffolds with a scaffold N50 of 21.0 Mb ( Table 1). The snail plot in Figure 2 provides a summary of the assembly statistics, while the distribution of assembly scaffolds on GC proportion and coverage is shown in Figure 3. The cumulative assembly plot in Figure 4 shows curves for subsets of scaffolds assigned to different phyla. Most (99.31%) of the assembly sequence was assigned to 32 chromosomal-level scaffolds, representing 30 autosomes and the Z and W sex chromosomes. Chromosome-scale scaffolds confirmed by the Hi-C data are named in order of size ( Figure 5; Table 2). The W chromosome could not be scaffolded, as the Hi-C data were from a male specimen. While not fully phased, the assembly deposited is of one haplotype. Contigs corresponding to the second haplotype have also been deposited. The mitochondrial genome was also assembled and can be found as a contig within the multifasta file of the genome submission.

**Table 1.** Genome data for *Spicauda simplicius*, ilUrbSimp4.1.

Project accession data		
Assembly identifier	ilUrbSimp4.1	
Species	*Spicauda simplicius*	
Specimen	ilUrbSimp4	
NCBI taxonomy ID	355208	
BioProject	PRJEB60188	
BioSample ID	SAMEA111453820	
Isolate information	ilUrbSimp4, female: whole organism (PacBio DNA sequencing) ilUrbSimp8, male: whole organism (Illumina Hi-C sequencing)	
Assembly metrics *		*Benchmark*
Consensus quality (QV)	63.6	*≥ 50*
*k*-mer completeness	100.0%	*≥ 95%*
BUSCO **	C:98.4%[S:98.0%,D:0.4%], F:0.5%,M:1.1%,n:5,286	*C ≥ 95%*
Percentage of assembly mapped to chromosomes	99.31%	*≥ 95%*
Sex chromosomes	ZW	*localised homologous pairs*
Organelles	Mitochondrial genome: 15.54 kb	*complete single alleles*
Raw data accessions		
PacificBiosciences Sequel IIe	ERR10934064	
Hi-C Illumina	ERR10936402	
Genome assembly		
Assembly accession	GCA_949699795.1	
*Accession of alternate haplotype*	GCA_949699105.1	
Span (Mb)	610.1	
Number of contigs	939	
Contig N50 length (Mb)	1.2	
Number of scaffolds	172	
Scaffold N50 length (Mb)	21.0	
Longest scaffold (Mb)	27.66	
Genome annotation		
Number of protein-coding genes	18,506	
Number of gene transcripts	18,688	

* Assembly metric benchmarks are adapted from column VGP-2020 of “Table 1: Proposed standards and metrics for defining genome assembly quality” from Rhie *et al.* (2021). ** BUSCO scores based on the lepidoptera_odb10 BUSCO set using version v5.4.3. C = complete [S = single copy, D = duplicated], F = fragmented, M = missing, n = number of orthologues in comparison. A full set of BUSCO scores is available at https://blobtoolkit.genomehubs.org/view/Urbanus%20simplicius/dataset/ilUrbSimp4_1/busco.

**Table 2.** Chromosomal pseudomolecules in the genome assembly of *Spicauda simplicius*, ilUrbSimp4.

INSDC accession	Chromosome	Length (Mb)	GC%
OX453057.1	1	24.58	36.0
OX453058.1	2	24.56	36.0
OX453059.1	3	23.85	36.0
OX453060.1	4	22.96	36.0
OX453061.1	5	22.94	36.0
OX453062.1	6	22.9	36.0
OX453063.1	7	22.68	36.5
OX453064.1	8	22.43	36.0
OX453065.1	9	22.24	36.5
OX453066.1	10	22.23	36.0
OX453067.1	11	22.17	36.0
OX453068.1	12	21.78	36.0
OX453069.1	13	21.03	36.5
OX453070.1	14	20.63	36.0
OX453071.1	15	20.38	36.5
OX453072.1	16	20.25	36.5
OX453073.1	17	19.79	36.5
OX453074.1	18	18.59	36.5
OX453075.1	19	18.41	37.0
OX453076.1	20	18.03	37.0
OX453077.1	21	17.51	37.0
OX453078.1	22	16.41	37.5
OX453079.1	23	15.39	37.5
OX453080.1	24	15.1	38.0
OX453081.1	25	14.65	37.5
OX453082.1	26	14.17	38.0
OX453084.1	27	10.82	39.0
OX453085.1	28	10.43	38.5
OX453086.1	29	9.45	39.0
OX453087.1	30	9.21	39.5
OX453083.1	W	2.66	39.5
OX453056.1	Z	27.66	35.5
OX453088.1	MT	0.02	18.0

The estimated Quality Value (QV) of the final assembly is 63.6 with *k*-mer completeness of 100.0%, and the assembly has a BUSCO v5.4.3 completeness of 98.4% (single = 98.0%, duplicated = 0.4%), using the lepidoptera_odb10 reference set ( *n* = 5,286).

Metadata for specimens, BOLD barcode results, spectra estimates, sequencing runs, contaminants and pre-curation assembly statistics are given at https://links.tol.sanger.ac.uk/species/355208.

## Genome annotation report

The *Spicauda simplicius* genome assembly (GCA_949699795.1) was annotated at the European Bioinformatics Institute (EBI) on Ensembl Rapid Release. The resulting annotation includes 18,688 transcribed mRNAs from 18,506 protein-coding genes ( Table 1; https://rapid.ensembl.org/Urbanus_simplicius_GCA_949699795.1/Info/Index).

## Methods

### Sample acquisition and nucleic acid extraction

Specimens of *Spicauda simplicius* were collected from Tarapoto, San Martin, Peru (latitude –6.49, longitude –76.36) on 2021-10-01. The specimens were caught with a butterfly net in dense Amazonian forest. The specimens were collected by Daniel Linke (Biology Center CAS) and identified by Daniel Linke and Pavel Matos (Biology Center CAS) and preserved by freezing of whole organism. A specimen with ID SAN25000013 (ToLID ilUrbSimp4) was used for genome sequencing and a male specimen (specimen ID SAN25000016, ToLID ilUrbSimp8) was used for Hi-C scaffolding sequencing.

The workflow for high molecular weight (HMW) DNA extraction at the Wellcome Sanger Institute (WSI) Tree of Life Core Laboratory includes a sequence of core procedures: sample preparation; sample homogenisation, DNA extraction, fragmentation, and clean-up. In sample preparation, the ilUrbSimp4 sample was weighed and dissected on dry ice ( Jay *et al.*, 2023). Tissue from the whole organism was homogenised using a PowerMasher II tissue disruptor ( Denton *et al.*, 2023a). HMW DNA was extracted using the Manual MagAttract v1 protocol ( Strickland *et al.*, 2023b). DNA was sheared into an average fragment size of 12–20 kb in a Megaruptor 3 system with speed setting 30 ( Todorovic *et al.*, 2023). Sheared DNA was purified by solid-phase reversible immobilisation ( Strickland *et al.*, 2023a): in brief, the method employs a 1.8X ratio of AMPure PB beads to sample to eliminate shorter fragments and concentrate the DNA. The concentration of the sheared and purified DNA was assessed using a Nanodrop spectrophotometer and Qubit Fluorometer and Qubit dsDNA High Sensitivity Assay kit. Fragment size distribution was evaluated by running the sample on the FemtoPulse system.

Protocols developed by the WSI Tree of Life laboratory are publicly available on protocols.io ( Denton *et al.*, 2023b).

### Sequencing

Pacific Biosciences HiFi circular consensus DNA sequencing libraries were constructed according to the manufacturers’ instructions. DNA sequencing was performed by the Scientific Operations core at the WSI on a Pacific Biosciences Sequel IIe instrument. Hi-C data were also generated from whole organism tissue of ilUrbSimp8 using the Arima v2 kit. The Hi-C sequencing was performed using paired-end sequencing with a read length of 150 bp on the Illumina NovaSeq 6000 instrument.

### Genome assembly and curation

Assembly was carried out with Hifiasm ( Cheng *et al.*, 2021) and haplotypic duplication was identified and removed with purge_dups ( Guan *et al.*, 2020). The assembly was then scaffolded with Hi-C data ( Rao *et al.*, 2014) using YaHS ( Zhou *et al.*, 2023). The assembly was checked for contamination and corrected as described previously ( Howe *et al.*, 2021). Manual curation was performed using HiGlass ( Kerpedjiev *et al.*, 2018) and PretextView ( Harry, 2022). The mitochondrial genome was assembled using MitoHiFi ( Uliano-Silva *et al.*, 2023), which runs MitoFinder ( Allio *et al.*, 2020) or MITOS ( Bernt *et al.*, 2013) and uses these annotations to select the final mitochondrial contig and to ensure the general quality of the sequence.

### Final assembly evaluation

A Hi-C map for the final assembly was produced using bwa-mem2 ( Vasimuddin *et al.*, 2019) in the Cooler file format ( Abdennur & Mirny, 2020). To assess the assembly metrics, the *k*-mer completeness and QV consensus quality values were calculated in Merqury ( Rhie *et al.*, 2020). This work was done using Nextflow ( Di Tommaso *et al.*, 2017) DSL2 pipelines “sanger-tol/readmapping” ( Surana *et al.*, 2023a) and “sanger-tol/genomenote” ( Surana *et al.*, 2023b). The genome was analysed within the BlobToolKit environment ( Challis *et al.*, 2020) and BUSCO scores ( Manni *et al.*, 2021; Simão *et al.*, 2015) were calculated.

Table 3 contains a list of relevant software tool versions and sources.

**Table 3.** Software tools: versions and sources.

Software tool	Version	Source
BlobToolKit	4.2.1	https://github.com/blobtoolkit/blobtoolkit
BUSCO	5.3.2	https://gitlab.com/ezlab/busco
Hifiasm	0.16.1-r375	https://github.com/chhylp123/hifiasm
HiGlass	1.11.6	https://github.com/higlass/higlass
Merqury	MerquryFK	https://github.com/thegenemyers/MERQURY.FK
MitoHiFi	3	https://github.com/marcelauliano/MitoHiFi
PretextView	0.2	https://github.com/wtsi-hpag/PretextView
purge_dups	1.2.3	https://github.com/dfguan/purge_dups
sanger-tol/genomenote	v1.0	https://github.com/sanger-tol/genomenote
sanger-tol/readmapping	1.1.0	https://github.com/sanger-tol/readmapping/tree/1.1.0
YaHS	1.2a	https://github.com/c-zhou/yahs

### Genome annotation

The BRAKER2 pipeline ( Brůna *et al.*, 2021) was used in the default protein mode to generate annotation for the *Spicauda simplicius* assembly (GCA_949699795.1) in Ensembl Rapid Release at the EBI.

### Wellcome Sanger Institute – Legal and Governance

The materials that have contributed to this genome note have been supplied by a Darwin Tree of Life Partner. The submission of materials by a Darwin Tree of Life Partner is subject to the **‘Darwin Tree of Life Project Sampling Code of Practice’**, which can be found in full on the Darwin Tree of Life website here. By agreeing with and signing up to the Sampling Code of Practice, the Darwin Tree of Life Partner agrees they will meet the legal and ethical requirements and standards set out within this document in respect of all samples acquired for, and supplied to, the Darwin Tree of Life Project.

Further, the Wellcome Sanger Institute employs a process whereby due diligence is carried out proportionate to the nature of the materials themselves, and the circumstances under which they have been/are to be collected and provided for use. The purpose of this is to address and mitigate any potential legal and/or ethical implications of receipt and use of the materials as part of the research project, and to ensure that in doing so we align with best practice wherever possible. The overarching areas of consideration are:

• Ethical review of provenance and sourcing of the material

• Legality of collection, transfer and use (national and international) 

Each transfer of samples is further undertaken according to a Research Collaboration Agreement or Material Transfer Agreement entered into by the Darwin Tree of Life Partner, Genome Research Limited (operating as the Wellcome Sanger Institute), and in some circumstances other Darwin Tree of Life collaborators.

## Data Availability

European Nucleotide Archive: *Urbanus simplicius* (plain longtail butterfly). Accession number PRJEB60188; https://identifiers.org/ena.embl/PRJEB60188 ( Wellcome Sanger Institute, 2023). The genome sequence is released openly for reuse. All raw sequence data and the assembly have been deposited in INSDC databases. Raw data and assembly accession identifiers are reported in Table 1.

## Author information

Members of the Wellcome Sanger Institute Tree of Life Management, Samples and Laboratory team are listed here: https://doi.org/10.5281/zenodo.10066175.

Members of Wellcome Sanger Institute Scientific Operations: Sequencing Operations are listed here: https://doi.org/10.5281/zenodo.10043364.

Members of the Wellcome Sanger Institute Tree of Life Core Informatics team are listed here: https://doi.org/10.5281/zenodo.10066637.

Members of the Tree of Life Core Informatics collective are listed here: https://doi.org/10.5281/zenodo.5013541.
