# Supplementary material for: The genome sequence of the Plain Longtail butterfly, Spicauda simplicius (Stoll, 1807)
Source: Wellcome Open Res. 2024 Jun 13;9:314. [Version 1] doi: 10.12688/wellcomeopenres.22457.1 (PMC11377922; doi:10.12688/wellcomeopenres.22457.1)
Supplement: Peer review history

# Reviewer report: Reviewer response for version 1

**DOI:** 10.21956/wellcomeopenres.24744.r94772

**Published:** 2024-09-05

**Authors:** Jerome H L Hui

**Affiliations:** 1 The Chinese University of Hong Kong, Hong Kong, Hong Kong

In this data note, Ribeiro and colleagues obtained the genomic resource of a female plain longtail butterfly *Spicauda simplicius *(Lepidoptera; Hesperiidae). The author stated that *Spicauda simplicius *as (Stoll, 1807), while other studies have been referring *Spicauda simplicius *as (Stoll, 1790)(e.g. TAXREF v17.0, https://inpn.mnhn.fr/espece/cd_nom/985422). I have also tried to look up and dig into different information, nevertheless, I did not have access to some crucial studies during the draft of this report to rectify this issue. I hope the authors can ensure that they are using the right one, especially when many in the future will refer to this data note when using this genomic resource.

 Prior to this study, there are limited molecular data available for this species deposited on the NCBI database. As of August 2024, this species remains unassessed by IUCN. This genome resource is important and will be very useful for further studies, such as understanding the ecological, evolutionary, and genomics questions related to lepidopterans and other insects more widely.

 This genome resource is excellent according to the summary statistics, with high BUSCO number scores, high sequence continuity (scaffold N50), and majority of sequences contained on the 30 pseudochromosomes (plus 2 sex chromosomes and mitochondrion). All in all, this is a valuable contribution.

Are sufficient details of methods and materials provided to allow replication by others?

Yes

Is the rationale for creating the dataset(s) clearly described?

Yes

Are the datasets clearly presented in a useable and accessible format?

Yes

Are the protocols appropriate and is the work technically sound?

Yes

Reviewer Expertise:

Genomics, evolution, invertebrates

I confirm that I have read this submission and believe that I have an appropriate level of expertise to confirm that it is of an acceptable scientific standard.

# Reviewer report: Reviewer response for version 1

**DOI:** 10.21956/wellcomeopenres.24744.r93320

**Published:** 2024-08-26

**Authors:** Elena Pazhenkova

**Affiliations:** 1 University of Ljubljana, Ljubljana, Slovenia

The article presents a comprehensive genome assembly of the Plain Longtail butterfly, *Spicauda simplicius* (Lepidoptera, Hesperiidae), a species with a neotropical distribution from northern Mexico to northern Argentina. The authors constructed a high-quality genome assembly using data from Pacific Biosciences single-molecule HiFi long reads and Hi-C data. The resulting genome sequence spans 610 Mb assigned to 32 chromosomal pseudomolecules, representing 30 autosomes and the Z and W sex chromosomes. Additionally, the mitochondrial genome has been assembled and included as a contig in the genome assembly.

 Gene annotation and manual curation refined the assembly by correcting 97 missing joins or mis-joins and removing 21 haplotypic duplications, resulting in a highly accurate and well-resolved genome sequence. The study provides detailed taxonomic information, habitat preferences, and host plant interactions, enhancing the ecological and evolutionary context of the research.

 Overall, this article offers a valuable resource for researchers interested in the biology and genomics of *Spicauda simplicius*. The genome assembly presented in this study serves as a robust foundation for future investigations into the species' adaptation, population structure, and evolutionary history.

Are sufficient details of methods and materials provided to allow replication by others?

Yes

Is the rationale for creating the dataset(s) clearly described?

Yes

Are the datasets clearly presented in a useable and accessible format?

Yes

Are the protocols appropriate and is the work technically sound?

Yes

Reviewer Expertise:

Entomology, conservation, bioinformatics.

I confirm that I have read this submission and believe that I have an appropriate level of expertise to confirm that it is of an acceptable scientific standard.
